# Correction: Real-time, smartphone-based processing of lateral flow assays for early failure detection and rapid testing workflows

**DOI:** 10.1039/d3sd90022c

**Published:** 2023-11-07

**Authors:** Monika Colombo, Léonard Bezinge, Andres Rocha Tapia, Chih-Jen Shih, Andrew J. deMello, Daniel A. Richards

**Affiliations:** a Institute for Chemical and Bioengineering, ETH Zurich Vladimir-Prelog-Weg 1 8093 Zürich Switzerland andrew.demello@chem.ethz.ch daniel.richards@chem.ethz.ch

## Abstract

Correction for ‘Real-time, smartphone-based processing of lateral flow assays for early failure detection and rapid testing workflows’ by Monika Colombo *et al.*, *Sens. Diagn.*, 2023, **2**, 100–110, https://doi.org/10.1039/D2SD00197G.

The authors regret that a statement, confirming that informed consent was obtained from the human participants involved in this study, was omitted in error from the published article. The volunteers were recruited from the authors' research group, the results were completely anonymised, and no personal data (other than name) were collected during the process.

The Royal Society of Chemistry apologises for these errors and any consequent inconvenience to authors and readers.

## Supplementary Material

